# Supplementary material for: Tandem Electrospray Mass Spectrometry of Cyclic N-Substituted Oligo-β-(1→6)-D-glucosamines
Source: Int J Mol Sci. 2020 Nov 5;21(21):8284. doi: 10.3390/ijms21218284 (PMC7663939; doi:10.3390/ijms21218284)
Supplement: Supplementary file 1 [file ijms-21-08284-s001.pdf]

International Journal of *Molecular Sciences*

MDPI

Table S1. The most abundant ions in the electrospray high-resolution mass spectra (ESI HRMS) of *N*-acyl derivatives of cyclooligo-β-(1→6)-D-glucosamines **1 — 24.**

Comp.	Ion	*m/z*, experiment	*m/z*, calculated	References[1]
	$[M+H]^+$	483.1974	483.1973	
	$[M+NH_4]^+$	500.2242	500.2239	
**1**	$[M+Na]^+$	505.1792	505.1793	[1]
	$[M+K]^+$	521.1524	521.1532	
	$[M–H]^+$	481.1826	481.1828	
	$[M+H]^+$	724.2930	724.2923	
	$[M+NH_4]^+$	741.3194	741.3189	
**2**	$[M+Na]^+$	746.2751	746.2743	[1]
	$[M+K]^+$	762.2488	762.2482	
	$[M–H]^+$	722.2785	722.2778	
	$[M+H]^+$	965.3871	965.3874	
	$[M+NH_4]^+$	982.4137	982.4139	
	$[M+Na]^+$	987.3692	987.3693	
	$[M+K]^+$	1003.3429	1003.3432	
	$[M+2H]^{2+}$	483.1972	483.1973	
**3**	$[M+H+NH_4]^{2+}$	491.7106		[1]
	$[M+H+Na]^{2+}$	494.1859	491.1883	
	$[M+H+K]^{2+}$	502.1725	502.1753	
	$[M+2Na]^{2+}$		505.1793	
	$[M+Na+K]^{2+}$	513.1654	513.1662	
	$[M+2K]^{2+}$	521.1525	521.1532	
	$[M+H]^+$	779.4900	779.4900	
	$[M+NH_4]^+$	796.6164	796.5165	
**4**	$[M+Na]^+$	801.4717	801.4719	[2]
	$[M+K]^+$	817.4479	817.4459	
	$[M+2H]^{2+}$	390.2486	390.2486	
	$[M+H]^+$	1168.7280	1168.7313	
	$[M+NH_4]^+$	1185.7545	1185.7579	
	$[M+Na]^+$	1190.7099	1190.7133	
**5**	$[M+K]^+$	1206.6850	1206.6872	[2]
	$[M+2Na]^{2+}$	606.8502	606.8512	
	$[M+H+K]^{2+}$	603.8465	603.8472	
	$[M+2K]^{2+}$	622.8255	622.8252	
	$[M+Na]^+$	1579.9571	1579.9546	
	$[M+2H]^{2+}$	779.4873	779.4900	
	$[M+H+NH_4]^{2+}$	788.0004	788.0003	
**6**	$[M+H+Na]^{2+}$	790.4788	790.4810	[1]
	$[M+2NH_4]^{2+}$	796.5134	796.5165	
	$[M+2Na]^{2+}$	801.4704	801.4719	
	$[M+NH_4+K]^{2+}$	806.9790	806.9812	
	$[M+Na+K]^{2+}$	809.4587	809.4589	
	$[M+NH_4]^+$	1964.2375	1964.2406	
	$[M+H+NH_4]^{2+}$	982.6222	982.6239	
	$[M+H+Na]^{2+}$	985.1008	985.1016	
**7**	$[M+2NH_4]^{2+}$	991.1357	991.1372	[2]
	$[M+NH_4+Na]^{2+}$	993.6078	993.6149	
	$[M+2Na]^{2+}$	996.0933	996.0926	
	$[M+NH_4+K]^{2+}$	1001.6008	1001.6019	
	$[M+Na+K]^{2+}$	1004.0803	1004.0796	
	$[M+NH_4]^+$	1176.7560	1176.7575	
	$[M+Na]^+$	1181.7118	1181.7129	
**8**	$[M+K]^+$	1197.6860	1197.6869	[2]
	$[M+2H]^{2+}$	580.3690	580.3691	
	$[M+2Na]^{2+}$	602.3509	609.3511	

**9**	$[M+H]^+$	611.3022	611.3022	[2]
	$[M+NH_4]^+$	628.3284	628.3287	
	$[M+Na]^+$	633.2841	633.2841	
	$[M+K]^+$	649.2579	649.2581	
**10**	$[M+H]^+$	916.4495	916.4496	[2]
	$[M+NH_4]^+$	933.4763	933.4762	
	$[2M+Na+NH_4]^{2+}$	935.9542	935.9539	
	$[M+Na]^+$	938.4318	938.4316	
	$[M+K]^+$	954.4062	954.4055	
**11**	$[M+H]^+$	1221.5960	1221.5971	[2]
	$[M+NH_4]^+$	1238.6221	1238.6236	
	$[M+Na]^+$	1243.5782	1243.5790	
	$[M+K]^+$	1259.5518	1259.5530	
	$[M+2H]^{2+}$	611.3022	611.3022	
	$[M+H+NH_4]^{2+}$	619.8146	619.8155	
	$[M+H+Na]^{2+}$	622.2924	622.2932	
	$[M+2NH_4]^{2+}$	628.3281	628.3287	
	$[M+H+K]^{2+}$	630.2766	630.2801	
	$[M+Na+NH_4]^{2+}$	630.7812	630.8064	
	$[M+2Na]^{2+}$	633.2831	633.2841	
	$[M+Na+K]^{2+}$	641.2705	641.2711	
	$[M+2K]^{2+}$	649.2572	649.2581	
**12**	$[M+2H]^{2+}$	763.8758	763.8759	[2]
	$[M+H+NH_4]^{2+}$	772.3887	772.3892	
	$[M+H+Na]^{2+}$	774.8662	774.8669	
	$[M+2NH_4]^{2+}$	780.9020	780.9025	
	$[M+H+K]^{2+}$	782.8511	782.8538	
	$[M+2Na]^{2+}$	785.8572	785.8579	
	$[M+Na+K]^{2+}$	793.8439	793.8448	
**13**	$[M+H]^+$	935.4073	935.4078	[2]
	$[M+NH_4]^+$	952.4339	952.4344	
	$[M+Na]^+$	957.3890	957.3898	
	$[M+K]^+$	973.3629	973.3637	
	$[M+2H]^{2+}$	468.2071	468.2076	
	$[M+H+Na]^{2+}$	479.1975	479.1985	
	$[M+H+K]^{2+}$	487.1815	487.1855	
	$[M+2Na]^{2+}$	490.1888	490.1895	
	$[M+Na+K]^{2+}$	498.1745	498.1765	
**14**	$[M+H]^+$	1157.4956	1157.4944	[1]
	$[M+NH_4]^+$	1174.5217	1174.5209	
	$[M+Na]^+$	1179.4770	1179.4763	
	$[M+K]^+$	1195.4510	1195.4502	
	$[M+2H]^{2+}$	579.2500	579.2508	
	$[M+H+NH_4]^{2+}$	587.7629	587.7641	
	$[M+H+Na]^{2+}$	590.2411	590.2418	
	$[M+H+K]^{2+}$	598.2275	598.2288	
	$[M+2Na]^{2+}$	601.2324	601.2328	
	$[M+Na+K]^{2+}$	609.2193	609.2197	
	$[M+2K]^{2+}$	617.2081	617.2067	
**15**	$[M+Na]^+$	1503.5794	1503.5819	[1]
	$[M+2H]^{2+}$	741.3032	741.3036	
	$[M+H+NH_4]^{2+}$	749.8165	749.8169	
	$[M+H+Na]^{2+}$	752.2940	752.2946	
	$[M+H+K]^{2+}$	760.2800	760.2816	
	$[M+2Na]^{2+}$	763.2851	763.2856	
	$[M+Na+K]^{2+}$	771.2720	771.2726	
	$[M+2K]^{2+}$	779.2590	779.2595	
**16**	$[M+Na]^+$	1503.5810	1503.5819	[1]
	$[M+2H]^{2+}$	741.3040	741.3036	
	$[M+H+NH_4]^{2+}$	749.8172	749.8169	
	$[M+H+Na]^{2+}$	752.2942	752.2946	

	$[M+H+K]^{2+}$	760.2793	760.2816	
	$[M+2Na]^{2+}$	763.2857	763.2856	
	$[M+Na+K]^{2+}$	771.2730	771.2726	
	$[M+2K]^{2+}$	779.2590	779.2595	
**17**	$[M+NH_4]^+$	1557.6502	1667.6511	[1]
	$[M+Na]^+$	1552.6062	1562.6065	
	$[M+K]^+$	1578.5797	1578.5804	
	$[M+H+NH_4]^{2+}$	779.3293	779.3292	
	$[M+H+Na]^{2+}$	781.8044	781.8069	
	$[M+2NH_4]^{2+}$	787.8429	787.8425	
	$[M+H+K]^{2+}$	789.7902	789.7938	
	$[M+2Na]^{2+}$	792.7980	792.7979	
	$[M+NH_4+K]^{2+}$	798.3075	798.3071	
	$[M+Na+K]^{2+}$	800.7844	800.7848	
**18**	$[M+NH_4]^+$	2070.8568	2070.8568	[1]
	$[M+H+NH_4]^{2+}$	1035.9293	1035.9321	
	$[M+H+Na]^{2+}$	1038.4035	1038.4098	
	$[M+2NH_4]^{2+}$	1044.4464	1044.4453	
	$[M+H+K]^{2+}$	1046.3938	1046.3967	
	$[M+NH_4+Na]^{2+}$	1046.9130	1046.9230	
	$[M+2Na]^{2+}$	1049.4046	1049.4007	
	$[M+NH_4+K]^{2+}$	1054.9108	1054.9100	
	$[M+Na+K]^{2+}$	1057.3876	1057.3877	
	$[M+2K]^{2+}$	1065.3754	1065.3747	
	$[M+3Na]^{3+}$	707.2671	707.2636	
**19**	$[M+NH_4]^+$	1756.1204	1756.1194	[3,4]
	$[M+Na]^+$	1761.0732	1761.0748	
	$[M+K]^+$	1777.0535	1777.0487	
	$[M+2H]^{2+}$	870.0502	870.0501	
	$[M+H+NH_4]^{2+}$	878.5637	878.5633	
	$[M+H+Na]^{2+}$	881.0416	881.0410	
	$[M+2NH_4]^{2+}$	887.0767	887.0766	
	$[M+H+K]^{2+}$	889.0310	889.0280	
	$[M+2Na]^{2+}$	892.0330	892.0320	
	$[M+NH_4+K]^{2+}$	897.5424	897.5413	
	$[M+Na+K]^{2+}$	900.0201	900.0190	
	$[M+2K]^{2+}$	908.0088	908.0060	
**20**	$[M+2NH_4]^{2+}$	1176.7567	1176.7575	[3]
	$[M+NH_4+Na]^{2+}$	1179.2311	1179.2352	
	$[M+2Na]^{2+}$	1181.7173	1181.7129	
	$[M+NH_4+K]^{2+}$	1187.2209	1187.2222	
	$[M+Na+K]^{2+}$	1189.7055	1189.6999	
	$[M+2K]^{2+}$	1197.6880	1197.6869	
	$[M+3H]^{3+}$	773.4887	773.4898	
	$[M+2H+NH_4]^{3+}$	779.1642	779.1653	
	$[M+H+2NH_4]^{3+}$	784.8400	784.8408	
**21**	$[M+2H]^{2+}$	1449.4095	1449.4119	[3,4]
	$[M+H+NH_4]^{2+}$	1457.9229	1457.9252	
	$[M+H+Na]^{2+}$	1460.4029	1460.4029	
	$[M+2NH_4]^{2+}$	1466.4364	1466.4385	
	$[M+H+K]^{2+}$	1468.3877	1468.3899	
	$[M+2Na]^{2+}$	1471.3955	1471.3939	
	$[M+NH_4+K]^{2+}$	1476.9029	1476.9031	
	$[M+Na+K]^{2+}$	1479.3853	1479.3808	
	$[M+3K]^{3+}$	988.5929	988.5923	
**22**	$[M+NH_4]^+$	1500.8640	1500.8632	[3]
	$[M+Na]^+$	1505.8264	1505.8186	
	$[M+2H]^{2+}$	742.4224	742.4220	
	$[M+H+NH_4]^{2+}$	750.9357	750.9352	
	$[M+H+Na]^{2+}$	753.4132	753.4129	
	$[M+2NH_4]^{2+}$	759.4487	759.4485	

	$[M+H+K]^{2+}$	761.3994	761.3999	
	$[M+2Na]^{2+}$	764.4047	764.4039	
	$[M+NH_4+K]^{2+}$	769.9136	769.9132	
	$[M+Na+K]^{2+}$	772.3911	772.3909	
**23**	$[M+NH_4]^{+}$	1953.8870	1953.8870	[1]
	$[M+Na]^{+}$	1958.8454	1958.8424	
	$[M+2NH_4]^{2+}$	985.9609	985.9604	
	$[M+NH_4+Na]^{2+}$	988.4344	988.4381	
	$[M+2Na]^{2+}$	990.9162	990.9158	
	$[M+Na+K]^{2+}$	998.9027	998.9028	
	$[M+3NH_4]^{3+}$	663.3190	663.3182	
	$[M+2H+2K]^{4+}$	503.9442	503.9485	
**24**	$[M+2H]^{2+}$	758.2723	758.2727	[1]
	$[M+H+NH_4]^{2+}$	766.7855	766.7859	
	$[M+H+Na]^{2+}$	769.2634	769.2636	
	$[M+H+K]^{2+}$	777.2498	777.2506	
	$[M+2Na]^{2+}$	780.2543	780.2546	
	$[M+NH_4+K]^{2+}$	785.7648	785.7639	
	$[M+Na+K]^{2+}$	788.2412	788.2416	
	$[M+2K]^{2+}$	796.2288	796.2286	

References

1. Gening M. L.; Titov D. V.; Cecioni S.; Audfray A.; Gerbst A. G.; Tsvetkov Y.E.; Krylov V.B.; Imberty A.; Nifantiev N. E.; Vidal S. Synthesis of multivalent carbohydrate-centered glycoclusters as nanomolar ligands of the bacterial lectin LecA from *Pseudomonas aeruginosa*. *Chem. Eur. J.*; **2013**, 19, 9272—9285.
2. Roy A.; Saha T.; Gening M. L.; Titov D. V.; Gerbst A. G.; Tsvetkov Y. E.; Nifantiev N. E.; Talukdar P. Trimodal control of ion-transport activity of cyclo-oligo-(1→6)-β-D-glucosamine-based artificial ion-transport systems. *Chem. Eur. J.*; **2015**, 21, 17445—17452.
3. Saha T.; Roy A.; Gening M.L.; Titov D.V.; Gerbst A.G.; Tsvetkov Y.E.; Nifantiev N.E.; Talukdar P. Cyclo-oligo-(1→6)-β-D-glucosamine based artificial channels for tunable transmembrane ion transport. *Chem. Commun*. **2014**, 50, 5514—5516.

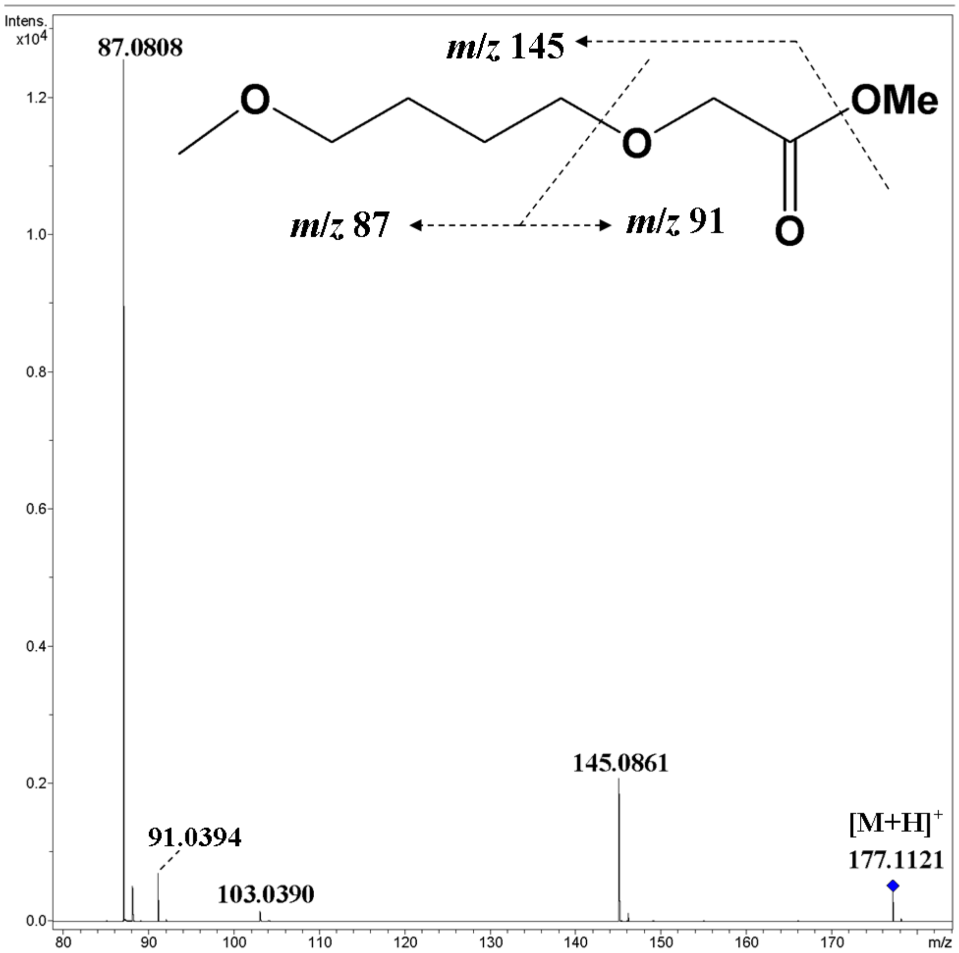

Figure 1. CID MS/MS of the $[M+H]^+$ ion of model compound $MeOCH_2CH_2CH_2CH_2OCH_2(CO)OMe$ and a scheme of its fragmentation (inset).
